# Endoscopic therapy for multifocal early esophageal cancer complicated by esophageal varices: a case report

**DOI:** 10.1093/gastro/goag038

**Published:** 2026-04-21

**Authors:** Yanbin Wei, Zinan Zhang, Jiafei Peng, Fuliang He, Lixue Xu, Xin Yao, Peng Li, Jie Xing

**Affiliations:** Department of Gastroenterology, Beijing Friendship Hospital, Capital Medical University, Beijing, P. R. China; State Key Laboratory of Digestive Health, Beijing, P. R. China; National Clinical Research Center for Digestive Disease, Beijing, P. R. China; Beijing Key Laboratory of Early Gastrointestinal Cancer Medicine and Medical Devices, Beijing, P. R. China; Department of Gastroenterology, Beijing Friendship Hospital, Capital Medical University, Beijing, P. R. China; State Key Laboratory of Digestive Health, Beijing, P. R. China; National Clinical Research Center for Digestive Disease, Beijing, P. R. China; Beijing Key Laboratory of Early Gastrointestinal Cancer Medicine and Medical Devices, Beijing, P. R. China; Department of Gastroenterology, Beijing Friendship Hospital, Capital Medical University, Beijing, P. R. China; State Key Laboratory of Digestive Health, Beijing, P. R. China; National Clinical Research Center for Digestive Disease, Beijing, P. R. China; Beijing Key Laboratory of Early Gastrointestinal Cancer Medicine and Medical Devices, Beijing, P. R. China; Department of Hepatology, Beijing Friendship Hospital, Capital Medical University, Beijing, P. R. China; Department of Imaging, Beijing Friendship Hospital, Capital Medical University, Beijing, P. R. China; Department of Gastroenterology, Beijing Friendship Hospital, Capital Medical University, Beijing, P. R. China; State Key Laboratory of Digestive Health, Beijing, P. R. China; National Clinical Research Center for Digestive Disease, Beijing, P. R. China; Beijing Key Laboratory of Early Gastrointestinal Cancer Medicine and Medical Devices, Beijing, P. R. China; National Clinical Research Center for Digestive Disease, Beijing, P. R. China; Department of Gastroenterology, Beijing Friendship Hospital, Capital Medical University, Beijing, P. R. China; State Key Laboratory of Digestive Health, Beijing, P. R. China; Beijing Key Laboratory of Early Gastrointestinal Cancer Medicine and Medical Devices, Beijing, P. R. China

## Introduction

Early esophageal cancer (EEC) is defined as a lesion confined to the mucosa or submucosa without lymph node metastasis [1]. Most EEC patients present with non-specific or absent symptoms. Endoscopic submucosal dissection (ESD) has become the standard treatment for EEC [2, 3]. EEC patients complicated by esophageal varices (EV) are generally excluded from ESD due to bleeding risks from submucosal variceal injury during deep dissection [4]. Most previous reports have focused on single-lesion EEC complicated by EV, typically employing endoscopic variceal ligation (EVL), endoscopic cyanoacrylate injection (ECI), or transjugular intrahepatic portosystemic shunt (TIPS) to reduce bleeding risk before proceeding with endoscopic resection [5, 6]. However, no guideline currently addresses the management of complex EEC complicated by EV, particularly in cases with multifocal lesions distributed across different esophageal quadrants and partially overlying varices. This case highlights the use of a staged and multimodal treatment approach.

## Case presentation

A 58-year-old male was diagnosed with alcohol-induced liver cirrhosis and EV (Child-Pugh A). An endoscopy revealed multifocal esophageal lesions with varices. Biopsy of one lesion showed squamous epithelial high-grade dysplasia with necrosis, suspicious for malignancy, while endoscopic ultrasound confirmed mucosal-layer confinement. Given the risks of invasion and metastasis, ESD was performed at an outside hospital before the initial EVL, but was discontinued due to a high intraprocedural bleeding risk. Only the lesion at 24 cm from the incisors was resected, anatomically distant from EV. Postoperative pathology confirmed both high- and low-grade squamous intraepithelial neoplasia.

Upon admission, the patient exhibited stable vital signs and unremarkable physical examination findings. Laboratory tests revealed no significant abnormalities in complete blood count, serum biochemistry, or coagulation profile. Contrast-enhanced abdominal computed tomography (CT) demonstrated features consistent with liver cirrhosis, including mild splenomegaly, portosystemic collateral formation, and splenorenal shunt.

**Figure 1:**
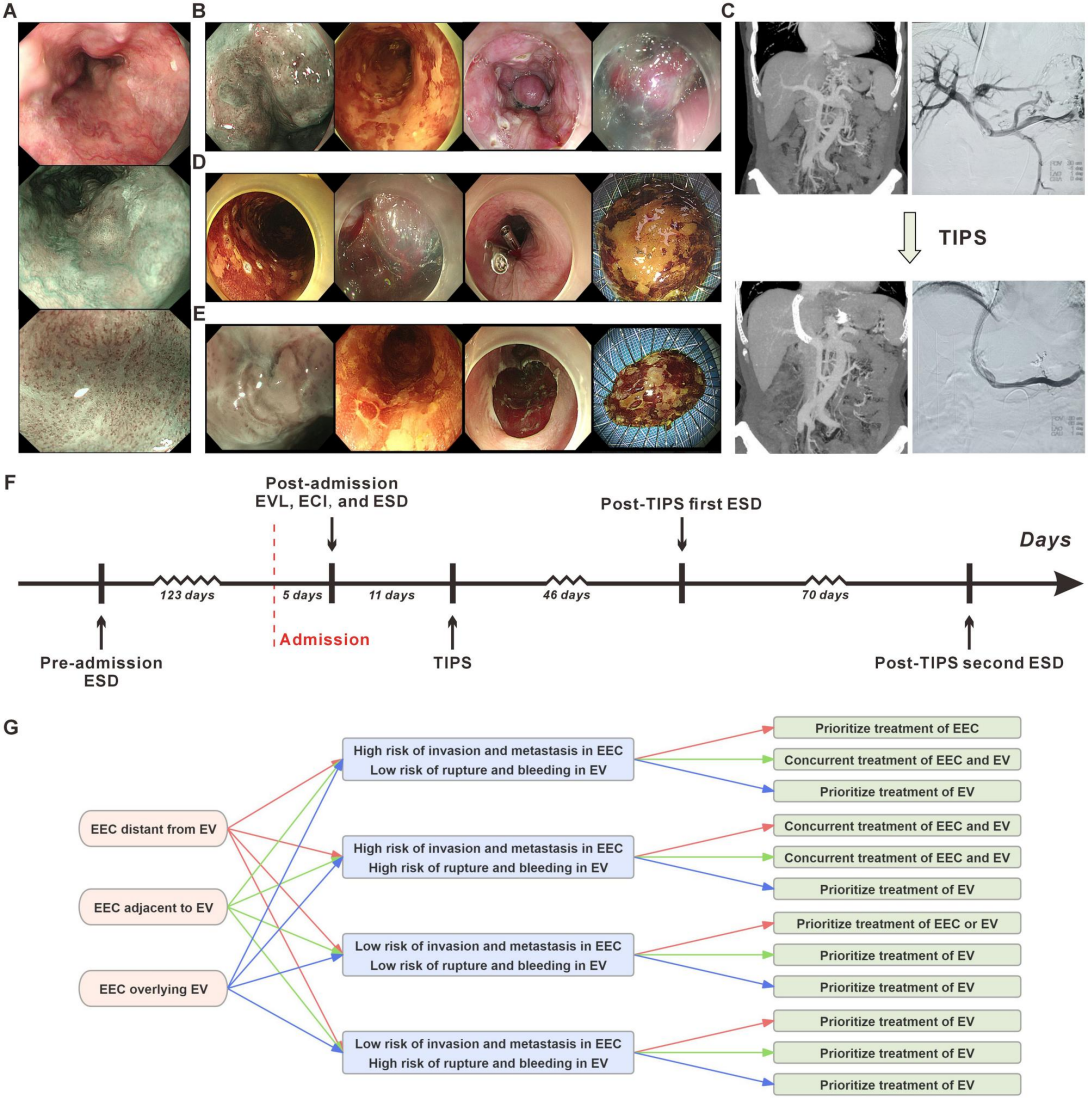
Images, treatment timeline, and specific situations of the treatment for patients with EEC complicated by EV. (A) White-light, narrow-band imaging, and magnified endoscopic images of mid-esophageal EEC complicated by EV. (B) Images of the endoscopic treatment before TIPS. (C) The abdominal contrast-enhanced CT and portal venography images before and after TIPS. (D) Images of the first endoscopic treatment after TIPS. (E) Images of the second endoscopic treatment after TIPS. (F) Detailed treatment timeline of the patient. (G) Structured risk assessment flowchart for EEC complicated by EV. Arrows of the same color indicate the specific treatment corresponding to the specific situation. ECI, endoscopic cyanoacrylate injection; EEC, early esophageal cancer; ESD, endoscopic submucosal dissection; EV, esophageal varices; EVL, endoscopic variceal ligation; TIPS, transjugular intrahepatic portosystemic shunt.

After admission, the patient underwent further evaluation with white-light endoscopy and magnifying endoscopy combined with narrow-band imaging (Fig. 1A). Multiple 0-IIb type lesions were detected at 27–28, 29–30, 33–36 and 34–37 cm from the incisors. Four blue varices were visible in the middle and lower segments of the esophagus, with some lesions overlying these varices. After excluding contraindications, the patient underwent endoscopic treatment (Fig. 1B and **Supplementary Video**). Two ligation points were applied through a Saeed multi-band ligator (MBL-6-F; Wilson-Cook Medical Incorporated, USA). The anal-side ligation point was placed at approximately 40 cm from the incisors, corresponding to the 2–4 o’clock position, which was about 1.5 cm from the target lesion located at 34–37 cm from the incisors, extending from the 6 o’clock to 12 o’clock positions. The oral-side ligation point was placed at approximately 39 cm from the incisors, corresponding to the 6–8 o’clock position, approximately 0.3 cm from the target lesion. After some of the varices turned white and shrank, ESD was performed to resect the lesions en bloc. During the procedure, bleeding from the varices adjacent to the resection site was observed. ECI was performed. With the “sandwich” technique, rapid sequential injection of 3 mL of 50% glucose, 1 mL of cyanoacrylate, and 1.5 mL of 50% glucose was performed through the bleeding point into the variceal lumen, and the adhesive was covered with the deeper wound surface. The remaining lesions were not treated. Postoperatively, the patient was managed with fasting, esomeprazole for acid suppression, octreotide for enzyme inhibition, cefotaxime for infection prophylaxis, and supportive fluid therapy.

About 10 days later, the patient underwent TIPS combined with gastroesophageal variceal embolization. Preoperative portal venous pressure measured 19 mmHg, which decreased to 14 mmHg post-intervention, with the portal pressure gradient significantly reduced from 13 mmHg to 3 mmHg. Postoperatively, levofloxacin was administered for infection prophylaxis, supplemented with L-ornithine-L-aspartate, lactulose, and rifaximin for hepatic encephalopathy prevention. Follow-up contrast-enhanced abdominal CT confirmed TIPS stent patency and demonstrated marked improvement in EV compared with preoperative findings (Fig. 1C).

During the following 4 months, the patient underwent two additional ESD to treat the residual lesions (Fig. 1D and E). All lesions were successfully completely resected. The patient did not report any significant discomfort. The treatment timeline for this case is shown in Fig. 1F.

## Discussion

Previous reports have predominantly described single-lesion EEC complicated by EV, with treatment strategies generally based on the principle of EV treatment followed by ESD [6]. The occurrence of multifocal EEC distributed across multiple esophageal quadrants and simultaneously overlying or adjacent to multiple EV is very rare. This case used a staged and multimodal treatment approach involving initial EVL and ECI with selective ESD of relatively safer lesions, followed by TIPS to further decompress the portal system, and ultimately two additional ESDs to achieve complete resection.

Based on these considerations, we propose a comprehensive management strategy for multifocal, multi-quadrant EEC with EV. Firstly, the guideline recommend that these patients be classified based on the Child-Pugh criteria before the treatment [7]. As shown in Fig. 1G, we have summarized some specific situations for EEC complicated by EV, which have been supported by a retrospective study [8]. Finally, postoperative management is equally crucial.

A case series showed that ESD was performed 1 month after TIPS, with no bleeding, infection, or hepatic encephalopathy [6]. EV significantly improves or even disappears approximately 1 month after EVL [9]. Interestingly, a study reported ESD was performed on the fourth day post-EVL [8]. In most cases, we suggest an interval of more than 1 month between EV treatment and ESD to ensure safety and efficacy, unless EEC carries a high risk of invasion or metastasis.

This case has several limitations. Due to the findings based on the treatment experience of a single patient, the generalizability of the conclusions is limited. In addition, this therapeutic approach is technically complex and resource-intensive, and may not be feasible in all centers. Its efficacy and safety need to be further validated in larger-scale, prospective studies.

## Conclusions

This case report describes a rare overlap of multifocal EEC with EV, highlighting the feasibility of a staged and multi-modal approach guided by the structured risk assessment flowchart. This strategy provides a practical guide for the personalized management of complex, overlapping lesions.

## Supplementary Material

goag038_Supplementary_Data
