# Supplementary material for: Endoscopic therapy for multifocal early esophageal cancer complicated by esophageal varices: a case report
Source: Gastroenterol Rep (Oxf). 2026 Apr 21;14:goag038. doi: 10.1093/gastro/goag038 (PMC13099638; doi:10.1093/gastro/goag038)
Supplement: goag038_Supplementary_Data [file goag038_supplementary_data.zip › Supplemental Material.docx]

**Supplemental Material**

**Supplemental Video.** The video footage of the endoscopic treatment before transjugular intrahepatic portosystemic shunt
